# Bayesian Inference of Nanoparticle-Broadened X-Ray Line Profiles

**DOI:** 10.6028/jres.109.012

**Published:** 2004-02-01

**Authors:** Nicholas Armstrong, Walter Kalceff, James P. Cline, John E. Bonevich

**Affiliations:** University of Technology Sydney, PO Box 123, Broadway NSW 2007, Australia; National Institute of Standards and Technology, Gaithersburg, MD 20899, USA

**Keywords:** Bayesian, fuzzy pixel, instrumental broadening, inverse problem, maximum entropy, morphology, nanoparticles, size broadening, size distribution, x-ray line profiles

## Abstract

A single-step, self-contained method for determining the crystallite-size distribution and shape from experimental x-ray line profile data is presented. It is shown that the crystallite-size distribution can be determined without invoking a functional form for the size distribution, determining instead the size distribution with the least assumptions by applying the Bayesian/MaxEnt method. The Bayesian/MaxEnt method is tested using both simulated and experimental CeO_2_ data, the results comparing favourably with experimental CeO_2_ data from TEM measurements.

## 1. Introduction

The analysis of x-ray line profile broadening can be considered as solving a series of inverse problems. There are usually two steps—removing the instrumental contribution (deconvolution), and determining the broadening contribution in terms of crystallite size and microstrain. Here we are concerned with quantifying only the size broadening, in terms of the shape and size distributions of the crystallites. We present a method that removes the instrumental broadening and determines the particle size distribution in a single step. The general theoretical framework developed makes it possible to determine the crystallite shape and average dimensions, and to fully quantify these results by also assigning uncertainties to them.

In general, there are two approaches that can be adopted. The first assumes functional forms for the size distribution and shape of the crystallites, and applies least squares fitting to determine the parameters defining the size distribution [1,2]. For pragmatic reasons, this approach is often used to ensure numerical stability; however, it is based on an explicit assumption for the crystallite size distribution and does not take into account the non-uniqueness of the solution.

The second approach takes into account the non-uniqueness of the problem of determining the size distribution *P*(***D***) from the experimental data, by assigning a probability to the solutions and enabling an average solution to be determined from the set of solutions; moreover, it also allows any *a priori* information and assumptions to be included and tested. This approach is embodied in the Bayesian and maximum entropy methods [3,4,5,6]. Essentially, Bayesian theory tells us how to express and manipulate probabilities. It might be said, therefore, that Bayesian theory helps us to ask the appropriate questions, while the maximum entropy method tells us how to assign values to quantities of interest.

## 2. X-Ray Line Profiles

### 2.1 Observed Profile

The observed line profile, *g*(2*θ*), can be expressed as

$$g(2\theta )=\int k(2\theta -2\theta^{\prime })f(2\theta^{\prime })d(2\theta^{\prime })+b(2\theta )+n(2\theta )$$ (1)

where *k*(2*θ*) defines the instrument profile and considers the imperfect optics of the diffractometer; *f*(2*θ*) is the specimen profile, which (apart from strain effects which are not covered here) characterizes the size broadening due to microstructural properties of the specimen (i.e., crystallite shape, distribution and dimensions); *b*(2*θ*) and *n*(2*θ*) are the background level and the noise distribution, respectively. The observed profile, Eq. (1), can also be expressed in terms of reciprocal-space units, *s*, centered about $s_{0}=\frac{2\sin\theta_{0}}{\lambda }$, as

$$g(s)=g(2\theta )\frac{d(2\theta )}{ds}$$ (2)

where $d(2\theta )=\frac{\lambda }{\cos\theta }ds$.

The problem we face is determining the size distribution and shape of the crystallites from Eq. (1), given our knowledge of the instrument kernel, *k*(2*θ*), and our understanding of the counting statistics, ***σ***^2^. We also want to quantify the specimen profile and size distribution by assigning error bars to them. Before addressing these questions, we review line profile broadening from nanocrystallites.

### 2.2 Crystallite-Size Broadening

**Fig. 1:**
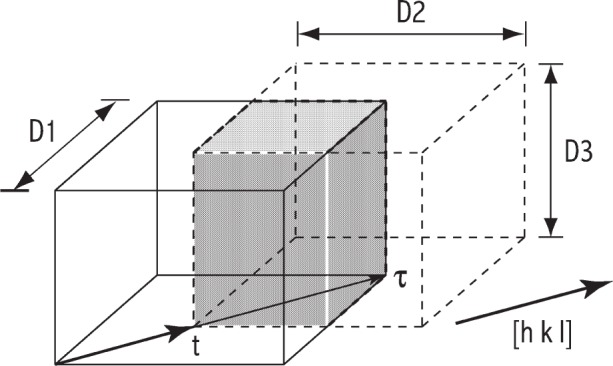
The crystallite (solid line) and its “ghost” (dashed line) shifted a distance *t* in the direction of the scattering vector [*hkl*]. The crystallite and ghost have dimensions ***D*** = {*D*_1_, *D*_2_, *D*_3_}. The shaded region represents the common volume between the crystallite and ghost. The maximum thickness of the crystallite in the direction [*hkl*] is *τ*. The common-volume function has the boundary conditions *V*(0, ***D***) = *V*_0_ and *V*(±*τ*, ***D***) = 0. As *t* → *τ*, *V*(*t*, ***D***) → 0. The plot of this function over *t* represents the Fourier coefficients from which the area- and volume-weighted sizes can be determined.

The line profile, *I*_p_(*s*,***D***), from a specimen consisting of crystallites of the same size and shape can be expressed in terms of the common-volume function [7] as

$$I_{p}(s,D)=2\int_{0}^{\tau }V(t,D)\cos\;2\pi \;s\;td\;t,$$ (3)

where *I*_p_(*s*,***D***) is the intensity profile given by the dimensions of the crystallite, ***D*** = {*D_i_*; *i* = 1,2,3}. The common-volume function of the crystallite, *V*(*t*, ***D***), quantifies the volume between the crystallite and its “ghost”, shifted a distance *t* parallel to the diffraction vector. The dimension *τ* represents the maximum length of the crystallite in the direction of the diffraction vector, and can be expressed in terms of the dimensions of the crystallite, ***D***, such that *τ* ≡ *τ* (***D***). The boundary conditions for the common-volume function are *V*(0, ***D***) = *V*_0_, where *V*_0_ is the volume of the crystal-lite, and *V*(±*τ*, ***D***) = 0. Figure 1 shows a schematic diagram of a crystallite and its ghost shifted a distance *t* in the direction [*hkl*]; the shaded region represents the common volume between the crystallite and its ghost. *V*(*t*, *D*) is symmetrical about the origin over the range *t* ∈ [–*τ*, *τ*]. This implies that *V*(*t*, ***D***) is an even function over this range. A simple example is a set of spherical crystallites with diameter *D*, for which the common-volume is given by [7] as

$$V(t,D)=\frac{\pi }{12}(t+2D){\left(t-D\right)}^{2}$$ (4a)

and using Eq. (3) the corresponding line-profile is [2,7]

$$I_{p}(s,D)=\frac{1}{16\pi^{3}s^{4}}+\frac{D^{2}}{8\pi s^{2}}-\frac{\cos(2\pi sD)}{16\pi^{3}s^{4}}-\frac{D\sin(2\pi sD)}{8\pi^{2}s^{3}}$$ (4b)

where *τ* (*D*) = *D* for spherical crystallites and in the limit of *s* → 0 [Eq. (4b)] reduces to *I*_p_(0, *D*) = π*D*^4^/8.

Essentially, Eq. (3) is the Fourier transform of the *V*(*t*, ***D***), and noting *V*(*t*, ***D***) is an even function, the odd (sine) terms in the Fourier transform vanish. This also implies that the size-broadened profiles will always be symmetrical about the Bragg angle, 2*θ*_0_. From Eq. (3) and Fig. 1, it is clear that information concerning the dimensions and shape of the crystallite is given in *V*(*t*, ***D***).

### 2.3 Particle-Size Distribution, *P*(*D*)

A powder specimen would not normally consist of crystallites all having the same size, but it can be assumed that the crystallites can have the same shape, based on kinetics arguments. The effect of the particle-size distribution on the common volume is to “blur” the broadening effects of a single crystallite.

The size-broadened line profile from a distribution of crystallites, P(D)DD, with dimensions in the range ***D*** to D+DD can be expressed as

$$f(s)=2\int_{0}^{\infty }\tilde{V}(t)\cos\;2\pi \;s\;td\;t,\;\forall s\in [-\infty ,+\infty ]$$ (5)

where $\tilde{V}(t)$ (*t*) is the *modified* common-volume function due to the influence of the particle-size distribution,

$$\tilde{V}(t)=\int_{t}^{\infty }V(t,D)P(D)DD.$$ (6)

In Eq. (6) a generalized measure, DD, has been used which is dependent on the crystallite shape and coordinate system. The area-weighted size, 〈*t*〉_a_, volume-weighted size, 〈*t*〉_v_, and column-length distribution (or area-weighted size distribution), *p*_a_(*t*), can be determined from Eq. (6) [8,9]. It can be seen from Eq. (6) how the shape and distribution of the crystallites influence the area- and volume-weighted quantities. Substituting Eq. (6) into Eq. (5), we have

$$f(s)=2\int_{t}^{\infty }\left[\int_{0}^{\infty }V(t,D)P(D)DD\right]\cos\;2\pi \;st\;dt$$ (7a)

$$=\int_{0}^{\infty }\left[2\int_{0}^{\tau }V(t,D)\cos\;2\pi \;st\;dt\right]P(D)DD,$$ (7b)

where in going from Eq. (7a) to Eq. (7b) the order of integration has been changed and *t* is integrated out. In addition we note that *V*(*t*, ***D***) ≥ 0 for *t* ∈ [0, *τ*] and *V*(*t*, ***D***) = 0 for *t* > *τ*. Inside the brackets of Eq. (7b), we have *I*_p_(*s*, ***D***) from Eq. (3). Hence, Eq. (7b) can be written as

$$f(s)=\int_{0}^{\infty }I_{p}(s,D)P(D)DD,\;\forall s\in [-\infty ,+\infty ]$$ (8)

where we define the profile kernel, *I*_p_(*s*, ***D***), as the size-broadened line profile given by a single crystallite with dimensions ***D***. In Eq. (8), we notice that the effect of *P*(***D***) is to weight the superposition of size profiles over the range of ***D*** to D+DD.

### 2.4 Determining *P*(*D*) From *g*(*s*)

In analysing the size distribution, we want to ensure that the statistics of the observed profile can be carried directly into quantifying the size distribution. Equation (8) expresses the specimen profile, *f*(*s*), in terms of the particle-size distribution and the shape of the nanocrystallites, while (1), after transformation into *s*-space, expresses the observed profile in terms of *f*(*s*). Combining these two equations, the experimental data, *g*(*s*), can be expressed in terms of the particle-size distribution, *P*(***D***) as

$$g(s)=\int_{0}^{+\infty }\int_{-\infty }^{+\infty }k(s-s^{\prime })I_{p}(s^{\prime },D)P(D)ds^{\prime }DD+b(s)+n(s)$$ (9a)

$$=\int_{0}^{+\infty }K(s,D)P(D)DD+b(s)+n(s)$$ (9b)

where the scattering kernel, *K*(*s*, ***D***), “rolls up” the instrumental effects and the profile kernel, and is given by

$$K(s,D)=\int_{-\infty }^{+\infty }k(s-s^{\prime })I_{p}(s^{\prime },D)ds^{\prime }.$$ (10)

In Eq. (10), the dummy variable *s*′ is being integrated out. The results given by Eqs. (9b) and (10) enable the particle-size distribution to be extracted directly from the experimental data. This ensures that the statistics of the experimental data are transferred to quantifying the uncertainty in the solution. This approach also addresses a difficulty of the *two-fold approach* discussed by Armstrong [10].

## 3. Bayesian and Maximum Entropy Methods

### 3.1 The Uniqueness of *P*(*D*)

In Eq. (9) we have a single expression for the observed profile in terms of the crystallite size distribution and shape, background level, and statistics of the experiment; information concerning the crystallite properties has been incorporated.

In seeking to determine *P*(***D***) from *g*(*s*), the issue of uniqueness for *P*(***D***) becomes important, for two reasons: firstly, because of the “conditioning” of the kernels, particularly *K*(*s*, ***D***); and secondly, due to the presence of statistical noise, ***σ***.

Generally, *K*(*s*, ***D***) will be *ill-conditioned*. This can be demonstrated in a numerical calculation by expressing *K*(*s*, ***D***) as a matrix, ***K***; we can show det***K***^T^***K*** ≈ 0. This implies that the column vectors of ***K*** are (nearly all) linearly dependent, which has dire consequences, as any attempt to determine *P*(***D***) (given *g*(*s*), *K*(*s*, ***D***), *σ* and *b*(*s*)), produces a set of solutions {*P*(*D*)} rather than a unique solution. The presence of statistical noise in the data simply worsens the situation, in that the ill-conditioning of *K*(*s*, ***D***) amplifies the noise and the solution is swamped by spurious and unphysical oscillations [11]. Faced with this situation, the following question arises:

How do we develop a method to extract a unique P(**D**) from g(s), given our knowledge of K(s, **D**), b(s) and σ^2^?

### 3.2 Some Observations

Before proceeding with developing a “method” to determine the crystallite size and shape from the observed data, *g*(*s*), some observations concerning these distributions need to be made.

The integral equations given by Eqs. (1) and (9) refer to a set of continuous functions. However, the recording of the observed and instrument profiles is made in discrete time intervals. To convey this, we express the observed profile, specimen profile and size distribution as vectors, such that ***g*** = {*g_i_*; *i* = 1, 2, 3, …, *M*}, ***f*** = {*f_j_*′; *j*′ = 1, 2, 3, …, *N*′} and ***P*** = {*P_j_*; *j* = 1, 2, 3, …, *N*}. The scattering kernel *K*(*s*, ***D***) can be expressed as a matrix, ***K*** = {*K_ij_*; ∀ *i*&*j*}, by taking the product of the instrument kernel and the line profile kernel. The instrument kernel can be evaluated in 2*θ*-space, such that ***R*** = {*k*(2*θ_i_* – 2*θ*′*_j_*_′_); *i* = 1, 2, 3, …, *M*&*j*′ = 1, 2, 3, …, *N*′}, and using $d(2\theta )=\frac{\lambda }{\cos\theta }ds$ can be mapped into *s*-space. Similarly, the profile kernel can be evaluated over *s* and ***D***, such that ***I***_p_ = {*I*_p_*_j_*_′_*_j_*; *j*′ = 1, 2, 3, …, *N*′ &*j* = 1, 2, 3, …, *N*}. The matrix product gives ***K*** = ***RI***_p_ and is an [*M* × *N*] matrix, such that *N* < *N*′ ≤ *M*.

There are *two* fundamental properties which *g*(2*θ*), *f*(2*θ*), *P*(***D***) and *V*(*t*, ***D***) all share. The first is that these distributions are *positive* definite; that is, the observed profile *g*(2*θ*) and specimen function *f*(2*θ*) represent intensities which are positive values. The second property is that these distributions are *additive*; that is, the sum of the distributions over a region represents a physically meaningful quantity [5]. For example, the integrated intensity of *g*(*s*) can be related back to the structure factor of the lattice, while the integrals ∫ *f* (*s*)d*s* and ∫*V*(*t*, ***D***)d*t* are inversely proportional to the integral breadth and quantify the specimen broadening in terms of size and strain contributions. The integral for *P*(***D***) is a special case, in that it must be unity. This ensures that we can attribute a probability for a particular ***D*** and determine its moments.

These two observations are important in formulating a “method” that can determine both the specimen profile from the observed x-ray diffraction profile and an underlying distribution such as the size distribution, *P*(***D***), while dealing with the issue of uniqueness. That is, we expect our method to extract this information from the observed data and produce results which preserve the positivity and additivity of the profile or distribution. It should also be possible to incorporate these properties of positivity and additivity without making additional assumptions about, say, the functional/analytical form of the specimen profile or size distribution. These conditions ensure that the specimen profile or size distribution determined from the observed profile can be interpreted in general terms.

In order to assign values to these distributions and preserve their additivity and positivity, a suitable function must be selected. Based on these observations and various arguments, the entropy function and maximum entropy principle are found to be the *only* consistent approach to inferring discrete probabilities (see [6,12,13,14,15,16,17]).

### 3.3 Bayes’ Theorem for *P*(*D*)

In analyzing size-broadened profiles, the central aim is to quantify the shape and size distribution of the crys-tallites, given the experimental data. Bayesian theory is well suited for testing a *hypothesis* in the presence of experimental data. This is achieved by quantifying the *a posteriori* probability distribution for ***P***, conditional on the experimental data and statistical noise. The formulation of Bayes’ theorem is general and can also be applied to determining ***f***.

Using Bayes’ theorem, the *a posteriori* probability for ***P*** is given by

$$\mathrm{Pr}(P|g,m,K,\sigma ,\alpha ,ℐ)=\frac{\mathrm{Pr}(P|m,\alpha ,ℐ)\mathrm{Pr}(g|P,K,\sigma ,ℐ)}{\mathrm{Pr}(g|m,K,\sigma ,ℐ)}$$ (11)

This is conditional on everything after ‘|’, viz., the observed profile ***g***, an *a priori* model ***m***, the scattering kernel ***K***, statistical noise *σ*, a constant *α*, and any additional background information concerning the experiment, *I*.

On the right-hand side of Eq. (11) there are several terms that require further discussion. The *likelihood* probability distribution Pr(***g*** | ***P***, ***K***, *σ*, *I*) defines the probability of measuring ***g***, given a size distribution ***P***, profile kernel ***K***, and statistical noise *σ*. That is, we include our hypothesis ***P***, and determine how probable it is to measure ***g***, given this hypothesis, ***K*** and ***σ***. The likelihood function is approximated as a Gaussian distribution for large counts (≫10) by applying the *central limit theorem*,

$$\mathrm{Pr}(g|P,K,\sigma ,ℐ)=\frac{1}{Z_{L}(\sigma )}\exp\left[-\frac{1}{2}L(P,g,K,\sigma ,)\right]$$ (12a)

where

$$L=\sum_{i=1}^{M}\frac{{\left(g_{i}-\sum_{j=1}^{N}K_{ij}P_{j}\right)}^{2}}{\sigma_{i}^{2}}$$ (12b)

and

$$Z_{L}(\sigma )=\prod_{i=1}^{M}\sqrt{2\pi \sigma_{i}^{2}}$$ (12c)

$$=\det\left\{\sqrt{2\pi \sigma^{2}}\right\},$$ (12d)

such that $\left\{\sqrt{2\pi \sigma^{2}}\right\}$ is an [*M* × *M*] diagonal matrix.

The variance is defined in terms of the observed counts and estimated background level as $\sigma_{i}^{2}=g_{i}+b_{i}^{\text{est}}$. In Eq. (12a), the kernel ***K*** has been included as it contains information about the shape of the crystallites and will influence the solution. We notice from Eq. (12b) that the matrix form of Eq. (9) has been incorporated.

The term Pr(P|m,α,ℐ) defines how probable is our hypothesis ***P***, given it is a positive and additive distribution and conditional on an *a priori* model, ***m***. The *a priori* probability distribution can be expressed as

$$\mathrm{Pr}(P|m,\alpha ,I)=\frac{1}{Z_{s}(\alpha )}\exp[\alpha ,S(P,m)].$$ (13a)

The entropy function is given as [6],

$$S(P,m)=\sum_{j=1}^{N}P_{j}-m_{j}-P_{j}\ln(P_{j}/m_{j}).$$ (13b)

where the normalization term, *Z_S_*(*α*) is given as

$$Z_{s}(\alpha )=\int DP\;\exp[\alpha \;S(P,m)]$$ (13c)

$$={\left(\frac{2\pi }{\alpha }\right)}^{\frac{N}{2}}$$ (13d)

$$=\frac{{\left(2\pi \right)}^{\frac{N}{2}}}{\sqrt{\det\;\alpha \;I}}$$ (13e)

and the integration in Eq. (13c) involves the measure $DP=\prod_{j=1}^{N}P_{j}^{-\frac{1}{2}}dP_{j}$. The log term in Eq. (13b) ensures that positive and additive distributions are obtained and that ***P*** will have these fundamental characteristics. The *a priori model*, ***m***, defines our ignorance/knowledge about ***P***. That is, if we are unsure of the shape of ***P***, it is best to admit our ignorance by assigning a uniform distribution over a specified range. The *a priori* model may also include data gathered from other sources, such as electron microscopy (e.g., TEM, SEM, and SPTM) techniques. It may also include theoretical or analytical models. For example, recently in the literature (see [1,2,18]) there has been a widespread use of the log-normal distribution for ***P***. However, in the Bayesian formulation we *do not explicitly* define ***P*** as a log-normal distribution, but set the *a priori* model as a log-normal distribution and test it in the presence of the observed data.

*S*(***P***, ***m***) is essentially a measure for ***P*** relative to ***m***. Suppose the model ***m*** was found to be a log-normal distribution and its parameters were determined using least squares analysis. If the resulting ***P*** lies “close” to ***m***, the change in *S* will be small; also, this would imply that the underlying crystallite-size distribution in the specimen is a log-normal distribution with values similar to those determined for ***m***, since this assumption has been tested in the presences of the experimental data. On the other hand, if ***P*** lies “some distance” from ***m***, the change in *S* will be large; this would imply that the underlying size distribution is not a log-normal distribution with the values estimated for ***m***.

The denominator term in Eq. (11) has an important application in selecting between various kernels, ***K***, for different crystallite shapes. It is called the *evidence* [4],

Pr(g|m,K,σ,ℐ)=∫DP∫dαPr(P,g,α|m,K,σ,ℐ). (14)

Including all the necessary terms, the *a posteriori* probability distribution for ***P*** can be expressed as

$$\mathrm{Pr}(P|g,m,K,\sigma ,\alpha ,ℐ)=\frac{1}{Z_{s}(\alpha )Z_{L}(\sigma )}\frac{e^{Q}}{\mathrm{Pr}(g|m,K,\sigma ,ℐ)}$$ (15)

where $Q=\alpha S-\frac{1}{2}L$. For convenience, *Q* ≡ *Q*(***P***, *α*), since ***P*** and *α* are the only two unknown terms. The *α* term in *Q*(***P***, *α*) can be interpreted as an undetermined Lagrangian multiplier.

Determining the most probable size distribution, $\hat{P}$, depends on maximizing Eq. (15), which in turn requires determining the global minimum for *Q*(***P***). There are several algorithms for determining $\hat{P}$ from *Q*(***P***), given its nonlinear characteristics (see [3,19]).

The approach we follow in determining the crystal-lite-size distribution is similar to that outlined by Bryan [3] and Jarrell and Gubernatis [20]. We start with a large *α* value and step towards *α* ≈ 0. For a given *α*, we determine ***P*** such that ∇*Q* = 0. After stepping through a range of *α* values, a set of solutions, {***P***(*α*)}, is formed parameterized by *α*. The average distribution, 〈***P***〉, can be determined from the set of solutions {***P***(*α*)},

$$\langle P\rangle =\int_{\alpha_{\min}}^{\alpha_{\max}}d\;\alpha \;P(\alpha )\mathrm{Pr}(\alpha |g,m,K,\sigma ,ℐ),$$ (16)

where Pr(α|g,m,K,σ,ℐ) is normalized to unity for *α* ∈ [*α*_min_, *α*_max_]. In the application of the Bayesian/MaxEnt method, the selected range was defined by *α* ∈ [10^–2^, 10^5^]. The average particle size distribution can be used to determine the average specimen profile, 〈*f*〉,

$$\begin{array}{l} \langle f\rangle =\int_{\alpha_{\min}}^{\alpha_{\max}}d\;\alpha \;I_{p}P(\alpha )\mathrm{Pr}(\alpha |g,m,K,\sigma ,ℐ)\\ =I_{p}\langle P\rangle . \end{array}$$

### 3.4 Determining Pr(α|g,m,K,σ,ℐ)

The *α* parameter in Eq. (15) is important in coupling the entropy function *S*(***P***, ***m***) with the likelihood function *L*(***P***). It is also a “nuisance parameter” and its influence can be integrated out. In evaluating Eq. (16), it is necessary to determine Pr(α|g,m,K,σ,ℐ); we do this by integrating out the ***P***,

$$\begin{array}{l} \mathrm{Pr}(\alpha |g,m,K,\sigma ,ℐ)=\int DP\mathrm{Pr}(P,\alpha |g,m,K,\sigma ,ℐ)\\ =\int DP\frac{\mathrm{Pr}(\alpha )|ℐ)\mathrm{Pr}(P|m,\alpha ,ℐ)\mathrm{Pr}(g|P,K,\sigma ,ℐ)}{\mathrm{Pr}(g|m,K,\sigma ,ℐ)}\\ =\frac{\mathrm{Pr}(\alpha |ℐ)}{\mathrm{Pr}(g|m,K,\sigma ,ℐ)}\frac{1}{Z_{s}(\alpha )Z_{L}(\sigma )}\\ \times \int DP\;e^{Q(P,\alpha )}, \end{array}$$ (17a)

and expanding $Q(P,\alpha )\approx Q(\hat{P},\alpha )+\frac{1}{2}{\left(P-\hat{P}\right)}^{T}\nabla \nabla Q(P-\hat{P})$ about $\hat{P}$ for a given *α*. We note ∇*Q* = 0 for $P=\hat{P}$ for a given *α*. On integrating, we have

$$\begin{array}{l} \mathrm{Pr}(\alpha |g,m,K,\sigma ,ℐ)\approx \frac{\mathrm{Pr}(\alpha |ℐ)}{\mathrm{Pr}(g|m,K,\sigma ,ℐ)}\frac{1}{Z_{s}(\alpha )Z_{L}(\sigma )}\\ \times \frac{{\left(2\pi \right)}^{Nq/2}e^{Q(\hat{P},\alpha )}}{\sqrt{\det\nabla \nabla Q(\alpha )}} \end{array}$$ (17b)

$$\begin{array}{l} =\frac{\mathrm{Pr}(\alpha |ℐ)}{\mathrm{Pr}(g|m,K,\sigma ,ℐ)}\frac{1}{Z_{L}(\sigma )}\\ \times \sqrt{\frac{\det\;\alpha \;I}{\det(\alpha \;I+\hat{\Lambda })}e^{Q(\hat{P},\alpha )}} \end{array}$$ (17c)

where $\nabla \nabla Q(\hat{P},\alpha )\equiv \nabla \nabla Q(\alpha )$ and $\hat{\Lambda }$ are the eigenvalues of ${\left(-\nabla \nabla S\right)}^{-\frac{1}{2}}\nabla \nabla L{\left(-\nabla \nabla S\right)}^{-\frac{1}{2}}=\left\{{\hat{P}}^{\frac{1}{2}}\right\}K^{T}\left\{\sigma^{-2}\right\}K\left\{{\hat{P}}^{\frac{1}{2}}\right\}$. The quantities in parentheses represent diagonal matrices. In Eq. (17a), we have introduced the *a priori* distribution for *α*, Pr(α|ℐ). Generally, we set Pr(α|ℐ) as a uniform model over a range [*α*_min_, *α*_max_]. Using Eq. (17) we can evaluate Eq. (16). In practice, we determine ln Pr(α|g,m,K,σ,ℐ) and $\hat{\Lambda }$ for each $\hat{P}$ and *α* in the range of [*α*_min_, *α*_max_].

### 3.5 Resolving Overlapped Profiles

The formalism presented here enables single and overlapped profiles, and even whole patterns, to be ana-lyzed, provided that crystallite-size effects are the major broadening component. Line profiles are generally overlapped due to low unit cell symmetry. However, specimen broadening, such as size broadening from crystallites, can also cause profiles to be overlapped. In this case, the underlying invariant quantity is the crystallite-size distribution, ***P***. The above integral equations for overlapped peaks can be expressed in terms of ***P***. The general form of Eq. (9) does not change; the term that does change is the kernel, ***K***(*s*, ***D***),

$$K(s,D)=\int_{-\infty }^{+\infty }\sum_{q}k(s-s^{\prime };{s^{\prime }}_{0_{q}})I_{p}(s^{\prime },D)ds^{\prime }$$ (18)

where ${s^{\prime }}_{0_{q}}=2\;\sin\;\theta_{0_{q}}/\lambda$ and $\theta_{0_{q}}$ is the Bragg angle at the *q*th peak in the pattern. The $k(s-s^{\prime };{s^{\prime }}_{0_{q}})$ term expresses the instrument kernel at each peak position, $\theta_{0_{q}}$. The *I_p_*(*s*′, ***D***) term is invariant over the range of *s*. In terms of the Bayesian analysis presented above, nothing else changes.

### 3.6 Error Analysis

Determining the errors in ***P*** over regions of importance is a final test for the quality of ***P***. The error bars for ***P*** are dependent on the choice of the *a priori* model and the quality of the observed data, *σ*.

It is only possible to assign error bars over a defined region, because the errors between points are strongly correlated [5,6]. The region of interest may consist of features in the specimen profile or size distribution which may not be physical, such as ripples in the tails of the distribution or a second peak suggesting a bimodal distribution. Over the defined region, we are interested in the *average integrated flux* [6],

$$\rho =\sum_{j=1}^{N}P_{j}w_{j}/\sum_{j=1}^{N}w_{j}w_{j}$$ (19)

$$=P^{T}w/w^{T}w$$ (20)

where ***w*** is a “window function” defined as,

$$w_{j}=\{\begin{array}{cc} 1 & r\leq j\leq r^{\prime },\\ 0, & \text{otherwise} \end{array}$$ (21)

and the region of interest is defined by *rr*′. Expanding Pr(P|g,m,K,σ,α,ℐ) about $\hat{P}$, we have $\mathrm{Pr}(P|g,m,K,\sigma ,\alpha ,ℐ)\propto e^{\frac{1}{2}{\left(P-\hat{P}\right)}^{T}\nabla \nabla Q\left(P-\hat{P}\right)}$. This is a Gaussian centered about $\hat{P}$. By inspection, the covariance matrix for ***P*** is given by –(∇∇*Q*)^–1^, where the elements in –(∇∇*Q*)^–1^ are strongly correlated with neighboring elements. Following the suggestion of Skilling [6] the variance for ***P*** is

$$\sigma_{P}^{2}=w^{T}\left[-{\left(\nabla \nabla Q\right)}^{-1}\right]w/w^{T}w.$$ (22)

Hence, we can assign error bars over a region of interest to the integrated flux of ***P***.

### 3.7 Fuzzy Pixel Approach for Determining *f*

It is often important to assess the specimen broadening by determining ***f***, without making any assumptions concerning its functional form. This can be achieved by deconvolving (1). However, in determining ***f*** “ringing effects” can appear in the solution. The ringing is often due to noise which are amplified and appear as unphysical oscillations in the solution (for example see Fig. 6 in [10]). The above theory assumes that smoothing is applied globally. However, the ringing effects are local artifacts. In order to introduce ‘local’ smoothing, we must address how to decompose ***f***. Explicit in the composition of ***f*** is that it is expressed as a superposition of delta functions,

$$f(2\theta )=\sum_{l=1}^{N}\delta (2\theta -2\theta_{l})a_{l}$$ (23)

where ***a*** = {*a*_1_, *a*_2_, …, *a*_N_} is the set of coefficients that define the amplitude of *f* at the *l*th position. Eq. (23) assumes a global smoothness, while the ringing effects are local effects.

Following the suggestion of Sivia [5,21], we blur *δ*(2*θ*) by including the spatial correlation length or width. To do this, we choose a basis function which includes a spatial correlation length as its width and reduces to *δ*(2*θ*) in the limit of the width going to zero. That is, we make the pixel at the *l*th position of ***f*** *fuzzy*. A simple choice is to express ***f*** in terms of a sum of Gaussian functions,

$$f(2\theta )=\sum_{l=1}^{N}\exp\left[-\frac{{\left(2\theta -2\theta_{l}\right)}^{2}}{2\omega^{2}}\right]a_{l}$$ (24)

where *ω* is the width of the spatial correlation or fuzzy pixel. In the limit of *ω* → 0, Eq. (24) reduces to Eq. (23).

In matrix notation Eq. (24) becomes,

f=Fa (25)

where ***F*** is an [*N* × *N*] matrix containing the elements of the Gaussian function.

How do we determine the optimum ω given the observed data, kernel and statistical noise?

The tools for addressing this question have been presented. That is, we employ Bayes’ theorem to determine the *a posteriori* probability distribution for *ω* conditional on the observed line profile. The *ω* that maximises the resulting *a posteriori* probability distribution becomes the optimum fuzzy pixel width, $\hat{\omega }$. At a practical level, we replace the equations where ***P*** appears with ***a***, and the kernel ***K*** is replaced by

G=RF (26)

where ***G*** ≡ ***G***(*ω*).

Applying Bayesian theory, the distribution for *ω* can be determined by integrating out ***a*** and *α*,

Pr(ω|g,m,σ,ℐ)=∫Da∫dαPr(a,α,ω|g,m,σ,ℐ) (27a)

$$\begin{array}{l} =\int Da\int d\;\alpha \mathrm{Pr}(a|ℐ)\mathrm{Pr}(\omega |ℐ)\\ \times \mathrm{Pr}(a|g,m,\sigma ,\alpha ,\omega ,ℐ). \end{array}$$ (27b)

Following the same steps as in Eq. (17), we have

$$\begin{array}{l} \mathrm{Pr}(\omega |g,m,\sigma ,ℐ)\approx \frac{\mathrm{Pr}(\alpha |ℐ)\;\mathrm{Pr}(\omega |ℐ)}{\mathrm{Pr}(g|m,\sigma ,ℐ)}\frac{1}{Z_{s}(\alpha )Z_{L}(\sigma )}\\ \;\times \frac{{\left(2\pi \right)}^{\frac{N}{2}}e^{Q(\alpha ,\omega )}}{\sqrt{\det\nabla \nabla Q(\alpha ,\omega )}}. \end{array}$$ (27c)

where *Q*(***a***, *α*, *ω*) = *αS*(***a***) – *L*(***a***, *ω*) for the unknown terms *a*, *α*, and *ω*; and ∇∇*Q*(*α*, *ω*) ≡ ∇*_a_*∇*_a_Q*(*α*, *ω*).

Error bars can also be attributed to ***a*** and ***f***. Using the results discussed in Sec. 3.6, the covariance matrix for ***a***, ∇*_a_*∇*_a_Q* can be determined. The corresponding covariance matrix for ***f*** can be determined from ∇*_f_*∇*_f_Q* = ***F***∇*_a_*∇*_a_Q****F****^T^*. On applying Eq. (22) the error bars for ***f*** can be determined.

Traditionally this problem has been solved by applying classical techniques, such as the Stokes method [22]. In order to overcome the numerical instability of the Stokes method, methods such as *direct convolution* [23,24] and profile fitting methods, such as the Voigt function [25,26,27,28,29] have been developed. These approaches assume an analytical function for the specimen profile; the convolution product between the instrument and specimen profile is refined (by updating the parameters that define the specimen profile) until the error between the calculated and observed data is minimized. These methods are a means to an end. There is often no physical basis for choosing a particular profile function, except that it results in a minimized error [30]. However, the Bayesian/fuzzy pixel/MaxEnt approach determines the *maximally uncommitted* solution or the solution with the least assumptions [31], given all the available data and information.

## 4. Generating and Analyzing Simulated CeO_2_ Data

### 4.1 Generating the Simulated Data

#### 4.1.1 Particle-Size Distribution, *P*(*D*)

In order to test the Bayesian/MaxEnt method, simulated data for the 200 and 400 line profiles from CeO_2_ were generated. The crystallites were assumed to be spherical in shape with a log-normal crystallite-size distribution,

$$P(D)=\frac{1}{\sqrt{2\pi D^{2}\ln^{2}\sigma_{0}}}\;\exp\;\left[-\frac{1}{2}{\left(\frac{\ln(D/D_{0})}{\ln\sigma_{0}}\right)}^{2}\right]$$ (28a)

where *D*_0_ is the median and $\sigma_{0}^{2}$ is the log-normal variance. The average diameter, 〈*D*〉, and variance, $\sigma_{\langle D\rangle }^{2}$, of the distribution are related to these quantities by

$$\langle D\rangle =D_{0}e^{\ln^{2}\sigma_{0}/2}$$ (28b)

and

$$\sigma_{\langle D\rangle }^{2}=D_{0}^{2}e^{\ln^{2}\sigma_{0}}\;\left(e^{\ln^{2}\sigma_{0}}-1\right)$$ (28c)

The log-normal parameters used were *D*_0_ = 13.03 nm and $\sigma_{0}^{2}=2.89$. Using Eqs. (28b and 28c), the average diameter and variance were determined to be, 〈*D*〉 = 15.00 nm and $\sigma_{\langle D\rangle }^{2}=73.17\;{\text{nm}}^{2}$, respectively. Using the results from Krill and Birringer [1] [see Eqs. (6)-(8), p. 625], the corresponding area- and volume-weighted sizes were determined.

The area- and volume-weighted diameters for spheres are related to the sizes [2] by

$${\langle D\rangle }_{a}=\frac{3}{2}{\langle t\rangle }_{a}$$ (29a)

and

$${\langle D\rangle }_{v}=\frac{4}{3}{\langle t\rangle }_{v}.$$ (29b)

The area- and volume-weighted sizes, 〈*t*〉_a_ and 〈*t*〉_v_, can be determined from the specimen profile, *f*, and Fourier coefficients, *A*(*t*), by using [32]

$${{\langle t\rangle }_{a}^{-1}=-\frac{dA(t)}{dt}|}_{t\to 0}.$$ (30)

The volume-weighted size is inversely related to the integral breadth and can be determined either directly from the specimen profile, *f*, or from its Fourier coefficients, *A*(*t*),

$$\beta =\int_{-\infty }^{\infty }f(s)ds/f_{\max}$$ (31a)

$$={\left[2\int_{0}^{\infty }A(t)dt\right]}^{-1}$$ (31b)

$$={\langle t\rangle }_{v}^{-1},$$ (31c)

where *β* is in reciprocal space units.

Using Eqs. (30) and (29), the area-weighted size and diameter were determined as 〈*t*〉_a_ = 17.56 nm and 〈*D*〉_a_ = 26.34 nm, respectively. Using Eqs. (31a) and (29), the volume-weighted size and diameter were determined as 〈*t*〉_v_ = 26.18 nm and 〈*D*〉_v_ = 34.91 nm, respectively. These settings are considered as the theoretical values for the simulated data. The Bayesian/fuzzy pixel/MaxEnt results were compared with the theoretical sizes, and percentage differences were determined.

#### 4.1.2 Line Profiles, *f*(2*θ*) and *k*(2*θ*)

Using the parameters for the size distribution, the specimen profile for spherical crystallites, *f*(2*θ*), was modelled over the range (2*θ*_0_ ± 10)°2*θ* at a step size of 0.01°2*θ* [see Eq. (8)]. The simulation of the specimen profile over this range minimized any artifacts in the Fourier coefficients. The instrument profile, *k*(2*θ*), was modelled on the diffractometer parameters and LaB_6_ line-position Standard Reference Material (SRM 660a), as discussed in Sec. 5.1. The split-Pearson VII function for the 200 line consisted of the following parameters: FWHM_low_ = 0.030°2*θ*, FWHM_high_ = 0.027°2*θ*, and *m*_exp,low_ = 6.928, *m*_exp,high_ = 11.324, where *m*_exp_ are the split-Pearson exponents. The “low” and “high” subscripts are with respect to the Bragg positions, 2*θ*_0_ (see Sec. 5.1).

#### 4.1.3 Generating *g*(2*θ*)

The observed line profiles, *g*(2*θ*), for the 200 and 400 lines consisted of the convolution of the specimen line profile, *f*(2*θ*), with the instrument line profile, *k*(2*θ*), Poisson noise, and a linear background level, *b*(2*θ*). Statistical noise was also imparted onto the background before adding it to the convoluted product. This is expressed by Eq. (1).

**Fig. 2:**
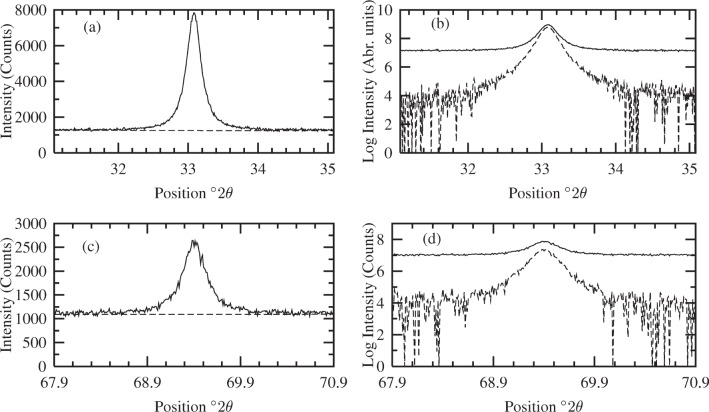
Simulated ‘observed’ 200 and 400 CeO_2_ profiles, *g*(2*θ*). (a) The 200 profile (solid line) and estimated background level (dashed line) over (2*θ*_0_ ± 2)°2*θ*, the range over which the analysis was carried out. (b) Logarithm of the 200 profile before (solid line) and after (dashed line) the background estimation. (c and d) Plots corresponding to (a) and (b), respectively, for the 400 profile over (2*θ*_0_ ± 1.5)°2*θ*.

The generation of *g*(2*θ*) was carried out over 2*θ*_0_ ± 10°2*θ* in order to minimize any truncation errors. The maximum peak height for the 200 line profile was set to 6500 counts (without background level and noise, or a total of 7835 counts including background level and noise) and the peak-to-background ratio, *R*_pb_, was set to 6.0. The corresponding percentage error in the peak maximum was determined using

$$\sigma_{\text{peak}}=\frac{1}{\left(R_{\text{pb}}-1\right)}{\left[\frac{R_{\text{pb}}(R_{\text{pb}}+1)}{I_{\max,\text{bg}}}\right]}^{\frac{1}{2}}\times 100\%$$ (32)

where *I*_max,bg_ is the maximum number of counts, including background level. Simulated *g*(2*θ*) for the 200 and 400 line profiles are shown in Fig. 2. The uncertainty for the 200 line was 1.5 % in the peak height. Similarly, for the 400 line the maximum peak height was set to 1500 counts (2646 counts including background level and noise); the average peak-to-background ratio was set to 2.4; and the estimated statistical uncertainty in the peak height was found to be 4.0 %.

In order to simulate realistic conditions, the Bayesian/MaxEnt analysis of the *g*(2*θ*) was carried out in a truncated region (2*θ*_0_ ± 2)°2*θ* for the 200 and (2*θ*_0_ ± 1.5)°2*θ* for the 400 line profiles. In the analysis, the background level was assumed to be unknown and was approximated by a linear function over this region. This was achieved by examining the Fourier coefficients of *g*(2*θ*) as the level was raised/lowered until distortions (i.e., “hook effect”, etc.) were removed. Figure 2 shows the simulated *g*(2*θ*) before and after the background level estimation for the 200 and 400 line profiles.

#### 4.1.4 Generating the Kernels, *R*, *I*_p_, and *K*

The numerical evaluation of the instrument kernel ***R***, line profile kernel ***I***_p_, and scattering kernel ***K***, are an important aspect in the application of the Bayesian/MaxEnt method. The evaluation of the fuzzy pixel kernel, ***F***, is also important in the implementation of the fuzzy pixel/MaxEnt method in determining the specimen profile, *f*. This section expands on Sec. 3.2.

The advantage of the Bryan algorithm [3] and the Bayesian/MaxEnt algorithm is that the search direction (or subspace) is defined by the singular value decomposition (SVD) of the scattering kernel, ***K***. This approach is numerically efficient (in that it reduces the number of floating point operations) and also numerically stable, since it does not utilize the full column-space of the kernels. As was pointed out in Sec. 3.2, the vector-space spanned by the column vectors of ***K*** may be all (or nearly all) linearly dependent, causing it to be ill-conditioned. The ill-conditioned characteristics are overcome by the SVD of ***K***, ***V***Σ***U****^T^*, where the “singular space” spanned by the column vectors of ***U*** is used to define the subspace in which the size distribution can be determined.

The instrument kernel, ***R***, is an [*M* × *N*′] matrix. The elements of this matrix can be determined by *R_ij_*_′_ = *k*(2*θ_i_* – 2*θ_j_*_′_), where *M* ≥ *N*′. This matrix can be mapped into reciprocal-space, *s*, by multiplying each column of ***R*** by d(2*θ*)/d*s* = *λ*/cos*θ_j_*_′_.

The line profile kernel expresses Eq. (3) as an [*N*′ × *N*] matrix, ***I***_p_ ≡ [***I***_p,_*_j_*_′_*_j_*], consisting of the line profile from a specific common volume (i.e., shape) function. The formalism presented here is completely general and any shape function can be used where appropriate. In this study, we have employed the common-volume function for spherical crystallites [see Eq. (4)],

$$I_{pj^{\prime }j}=\{\begin{array}{cc} \frac{1}{16\pi^{3}{s^{\prime }}_{j^{\prime }}^{4}}+\frac{D_{j}^{2}}{8\pi {s^{\prime }}_{j^{\prime }}^{2}}-\frac{\cos(2\pi {s^{\prime }}_{j^{\prime }}D_{j})}{16\pi^{3}{s^{\prime }}_{j^{\prime }}^{4}}-\frac{D_{j}\sin(2\pi {s^{\prime }}_{j^{\prime }}D_{j})}{8\pi^{2}{s^{\prime }}_{j^{\prime }}^{3}} & \text{for}\;{s^{\prime }}_{j^{\prime }}\neq 0\\ \frac{\pi D_{j}^{4}}{8} & \text{for}\;{s^{\prime }}_{j^{\prime }}=0 \end{array}$$ (33)

where the second term in Eq. (33) ensures that the line profile from a single spherical crystallite is finite for *s* = 0.

The evaluation of the scattering kernel, ***K***, is the matrix product of the instrument kernel (mapped into *s*-space), ***R***, and the line profile kernel, Eq. (33). Using Eq. (10),

$$K(s_{i},D_{j})=\delta D\delta s^{\prime }\sum_{j^{\prime }}k(s_{i}-{s^{\prime }}_{j^{\prime }})I_{p}({s^{\prime }}_{j^{\prime }},D_{j})$$ (34a)

$$K_{ij}=\delta D\delta s^{\prime }\sum_{j^{\prime }}R_{ij^{\prime }}I_{pj^{\prime }j}$$ (34b)

$$K=\delta D\delta s^{\prime }RI_{p}$$ (34c)

where ***R*** has been mapped into *s*-space, *δs*′ is the step size in *s*′-space and approximates the integration in Eq. (10), while *δD* is the step size in *D*-space and approximates the integration in Eq. (9). Care must be taken in selecting *δD* to avoid the under-sampling of Eq. (33).

### 4.2 Applying the Fuzzy Pixel/MaxEnt Method for *f*(2*θ*)

This approach involves determining the specimen profile from the simulated data. It is equivalent to solving the deconvolution problem, Eq. (1), and is an important first step in assessing the nature of the specimen broadening. In the past, we have applied the Skilling and Bryan [19] algorithm with global smoothing (see [5]), which we refer to here as the “old” MaxEnt method. However, in this section we apply the Fuzzy Pixel/MaxEnt method discussed in Sec. 3.7, to determine *f*(2*θ*). The results are also compared with those from the “old” MaxEnt method, and their reliability in reproducing the log-normal parameters for the crystallite-size distribution (specified in Sec. 4.1) is assessed.

**Fig. 3:**
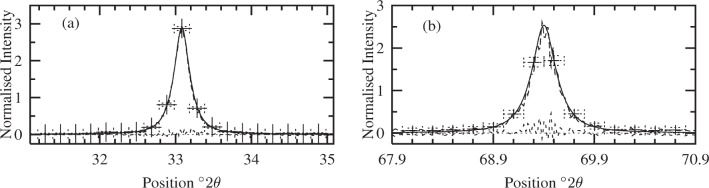
“Old” MaxEnt specimen profiles for the 200 and 400 line profiles. (a) The theoretical 200 specimen profile (solid line), “old” MaxEnt specimen profile (long dashed line + error bars) and the residuals (short dashed line). (b) Corresponding results for the 400 line profile, as shown in (a). The horizontal error bars in (a) and (b) represent non-overlapping region of interest, while the vertical error bars represent the uncertainty in the averaged integrated flux over the region of interest.

The specimen line profiles from the “old” MaxEnt approach are given in Fig. 3. These results were compared with the theoretical specimen profiles by evaluating the *R_f_* and *R_w_* values. A summary of these and subsequent analyses is given in Table 1.

**Fig. 4:**
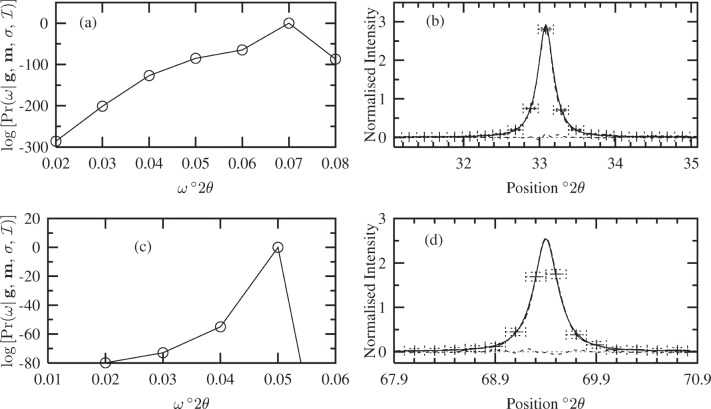
The fuzzy pixel distribution and MaxEnt solutions for the 200 and 400 line profiles. (a) The logPr(ω|g,m,σ,ℐ) distribution used to determine the optimum fuzzy pixel width, $\hat{\omega }\approx 0.07{}^{\circ}2\theta$ for the 200 specimen profile. (b) Theoretical specimen profile (solid line), fuzzy pixel/MaxEnt specimen profile (long dashed line + error bars) and the residuals (short dashed line) for the 200 line profile. (c) The logPr(ω|g,m,σ,ℐ) distribution used to determine the optimum fuzzy pixel width, $\hat{\omega }\approx 0.05{}^{\circ}2\theta$ for the 400 specimen profile. (d) Theoretical specimen profile (solid line), fuzzy pixel/MaxEnt specimen profile (long dashed line + error bars) and the residuals (short dashed line) for the 400 line profile. The horizontal error bars in (a) and (c) represent the non-overlapping region of interest, while the vertical error bar represents the uncertainty in the averaged integrated flux over the region of interest.

The “old” MaxEnt method is not based on a Bayesian formalism (see [4,6]) and spurious oscillations can appear in the solution specimen profile. This second point becomes important in analyzing high angle/low intensity profiles. This is further illustrated by inspecting the residuals in Fig. 3(b), where the amplitude of the residuals is large in comparison with the normalized peak height. We contrast the results in Fig. 3 with the fuzzy pixel/MaxEnt method discussed in Sec. 3.7. Using this theory, the fuzzy pixel distribution specimen profiles are shown in Fig. 4. The fuzzy pixel distribution determines the optimum fuzzy pixel width, *ω* [see Eq. (27)]. For the 200 line, the optimum value was found to be $\hat{\omega }\approx 0.07{}^{\circ}2\theta$ and for the 400 line, $\hat{\omega }\approx 0.05{}^{\circ}2\theta$. This defines the correlation-length scale of the noise in the simulated data and essentially filters out the noise effects. It is evident from the residuals of the multiple orders that smoothing of the specimen profile has been achieved using this approach.

Using the line profiles determined, and assuming a spherical crystallite shape, the parameters of the underlying log-normal size distribution can be reproduced by following the approach of Krill and Birringer [1]. These results are shown in Table 1. The analysis has produced mixed results, due to the stringent but realistic conditions imposed on the background estimation. Comparing the 200 line profile results for the “old” MaxEnt and fuzzy pixels methods, there is a noticeable improvement in the latter results over the former. This is not only seen in an improved *R_f_* value, but also in the reproduced log-normal parameters. In the case of the 400 line, we notice that the *R_f_* value has improved by a factor of ≈3 and the volume-weighted size by a factor of ≈1.5 for the fuzzy pixel/MaxEnt approach. However, the area-weighted size for the 400 line profile has not improved. As a consequence, when the underlying log-normal parameters are determined from the area- and volume-weighted sizes no improvements are gained.

These results for the 400 line profile can be explained by the low peak-to-background ratio, statistical uncertainty, and the presence of systematic errors arising from the background estimation. The peak-to-background ratio for the 400 line profile is 2.4. This low value results in an increased uncertainty in the estimated background level. From Eq. (32), we notice that as the peak-to-background ratio increases, the peak height uncertainty decreases and the dominant source of uncertainty becomes the statistical noise.^1^ The variance of the observed profile is determined by two components: the Poisson counting statistics, which can be approximated as $\sqrt{g}$, for *g* ≫ 10 counts, and the estimated background level, *b*_est_; it can be expressed as ***σ***^2^ = *g* + *b*_est_. The presence of statistical uncertainty and the low peak-to-background ratio introduces uncertainties to the slope and intercept of the estimated background level. In turn, this introduces systematic errors to the Fourier coefficients of *f* [30]. Although the fuzzy/pixel method has been successful in improving the quality of the line profile (which amounts to reducing the statistical error in the solution line profile), the systematic errors have propagated to the Fourier coefficients of the specimen profile and in turn to the area-weighted size. Additional calculations and applying the above analysis to simulated data with zero background (i.e., only Poisson noise) show percentage differences between the calculated and theoretical results of ≲ 5 % for both the 200 and 400 fuzzy pixel/MaxEnt specimen profiles. This highlights the difficulty of analyzing high-angle/weak line profiles, which clearly requires a good understanding of the background level in order to reduce the influence of systematic errors.

The application of the fuzzy pixel/MaxEnt method for determining *f*(2*θ*) enables the specimen broadening to be assessed. This is important in the application of methods such as those of Warren-Averbach and Williamson-Hall. Furthermore, the analysis discussed here can be used as the *a priori* information of the Bayesian/MaxEnt analysis. The fuzzy pixel/MaxEnt approach overcomes the difficulties in commonly-used deconvolution techniques (see [11]) and resolves the “ringing effects” in [10].

### 4.3 Bayesian/MaxEnt Method for *P*(*D*) Using Different *m*(*D*)

The next stage in the analysis of the simulated data is applying the Bayesian/MaxEnt method to determine the particle distribution, *P*(*D*). In addition, two different approaches for determining a model, *m*(*D*), were explored and their effects on *P*(*D*) were quantified. The two approaches were (i) uniform model over *D* ∈ [0, 60] nm and (ii) “low resolution” approach [30] using the log-normal distribution parameters determined in Sec. 4.2 as the prior.

#### 4.3.1 Uniform Model

**Fig. 5:**
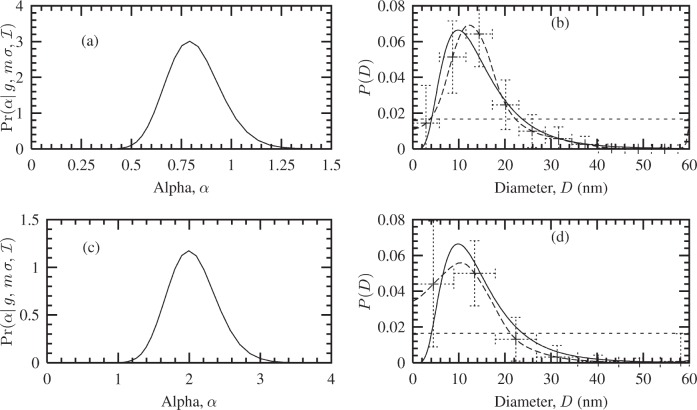
Bayesian/MaxEnt crystallite size distributions using a uniform *a priori* model. (a) The Pr(α|g,m,σ,ℐ) distribution, Eq. (17), used to average over the set of solutions, {***P***(*α*)}. (b) The theoretical crystallite size distribution (solid line), Bayesian/MaxEnt size distribution (long dashed line + error bars), and the uniform *a priori* model (short dashed line). (c) and (d) The corresponding Pr(α|g,m,σ,ℐ) distribution and Bayesian/MaxEnt size distribution for the 400 line profile.

The Fourier coefficients *A*(*t*) of the fuzzy pixel/MaxEnt specimen profiles (not shown here) suggest the maximum size of the crystallites is ≈ 60 nm, since *A*(*t*) ≈ 0 at this length. Using this information, a uniform distribution was defined over *D* ∈ [0, 60] nm. The corresponding Bayesian/MaxEnt results are shown in Fig. 5. The posterior distribution for *α* is shown in Figs. 5(a) and (c) for the 200 and 400 profiles, respectively. This distribution was used to average over the set of solutions {*P*} for each case. The Bayesian/MaxEnt results are given in Figs. 5(b) and (d) for the 200 and 400 profiles, respectively.

Using a uniform model, the Bayesian/MaxEnt size distributions where compared with the theoretical size distribution, *P*(*D*). The Bayesian/MaxEnt results share “global” features with the theoretical size distributions. However, “local” features are poorly defined, especially in the region of 0 < *D* < 10 nm. This is a direct consequence of the uniform model and the lack of relevant information in the data; that is, it assigns an equal weight to all sizes over *D*. The vertical error bars in both cases correctly represent the misfitting between the theoretical and Bayesian/MaxEnt size distributions; additionally, their magnitude also signifies that a uniform model transfers little or no useful information. This can also be seen in the parameters for the Bayesian/MaxEnt distribution compared with their theoretical values in Table 2. In determining the log-normal parameters from the Bayesian/MaxEnt *P*(*D*), the fitted distribution produces reasonable results. This suggests that, although the *a proiri* model is uniform, the Bayesian/MaxEnt method can “extract” some information concerning the underlying distribution from the simulated data.

#### 4.3.2 “Low Resolution” Approach

**Fig. 6:**
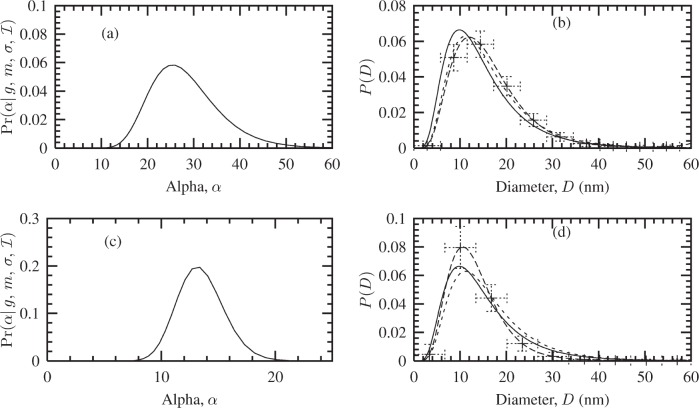
Bayesian/MaxEnt crystallite size distributions using a log-normal *a priori* model. (a) The Pr(α|g,m,σ,ℐ) distribution, Eq. (17), used to average over the set of solutions {***P***(*α*)}. (b) The theoretical crystallite size distribution (solid line), Bayesian/MaxEnt size distribution (long dashed line + error bars) and the low-resolution *a priori* model (short dashed line). (c) and (d) The corresponding Pr(α|g,m,σ,ℐ) distribution and Bayesian/MaxEnt size distribution for the 400 line profile.

A log-normal *a priori* model used in the Bayesian/MaxEnt method was defined from the *D*_0_ and *σ*_0_ of the 200 fuzzy pixel/MaxEnt line profile (see Table 1). Unlike the uniform model, this model defines local features of the size-distribution. The Bayesian/MaxEnt results using this model are shown in Fig. 6 and the determined parameters in Table 2. Before discussing the results, it is interesting to point out that the log-normal model and theoretical size-distribution produce a difference of 15.8 %. One of the aims of this section is to assess whether this difference has been imparted to the Bayesian/MaxEnt size-distribution.

Comparing the *a posteriori* distribution for *α* using a uniform model [see Figs. 5(a) and (c)] with that of the log-normal distribution, given in Figs. 6(a) and (c), we notice that the effect of the log-normal model is to shift the distribution in *α*-space and widen it. Essentially the solution space parameterized by *α* has been expanded to encompass those solutions which correspond to the available *a priori* and experimental data.

The Bayesian/MaxEnt size distributions, given in Figs. 6(b) and (d), compare reasonably well with the theoretical distribution. However, there is noticeable misfitting between these distributions. Further, the Bayesian/MaxEnt solution has been shifted slightly relative to the log-normal model. This is also evident in the *R_f_* for the 200 and 400 size distributions, given in Table 2. The *R_f_* for both solutions has increased relative to the log-normal model by an additional ≈3 % to 6 %. This can also be seen by comparing the percentage differences for the *D*_0_, *σ*_0_, 〈*D*〉, and $\sigma_{\langle D\rangle }^{2}$ parameters for the 200 fuzzy pixel solution, given in Table 1 (third column), with those given in Table 2 using the “low resolution” method, where there is a slight increase in the percentage difference, with the exception of the $\sigma_{\langle D\rangle }^{2}$ value. Additional calculations suggest that misfitting between the solution and theoretical size distributions arises from errors in the *a priori* model. The influence of the background estimation which was problematic in the fuzzy pixel analysis does not seem to be a factor in this analysis.

While there exists some misfitting between the solution and theoretical size distributions, the vertical error bars correctly account for this misfitting. This characteristic of the Bayesian/MaxEnt can be seen for both the uniform and non-uniform models. Indeed, this feature of the method ensures that it is fully quantitative, and represents a clear strength over existing methods. Comparing these solutions with those using a uniform model, considerable improvement in the size distribution has been achieved. The “local” information defined in the log-normal *a priori* model has been imparted to the Bayesian/MaxEnt solution.

This analysis also demonstrates the difficulty in estimating a suitable non-uniform model based on the current techniques. Further, any uncertainty in the model parameters is also passed on to the solution distribution. This indicates the need to quantify the uncertainty in the model parameters and quantify how these uncertainties are passed on to the solution size distribution.

## 5. Experimental Details

Analysis of the simulated data highlighted difficulties of background estimation and the effect of the *a priori* model on the Bayesian/MaxEnt size distribution. However, this analysis provided a useful understanding of the experimental condition which were used in conducting an appropriate set of measurements. The fuzzy pixel/Bayesian/MaxEnt methods were applied to experimental CeO_2_ diffraction data to determine the specimen profiles, crystallite shape, and size distribution. These results are compared with transmission electron microscopy data.

### 5.1 XRD Details

The CeO_2_ specimen used here was prepared for the CPD and IUCR size round robin by Louër and Audebrand [33].

**Fig. 7:**
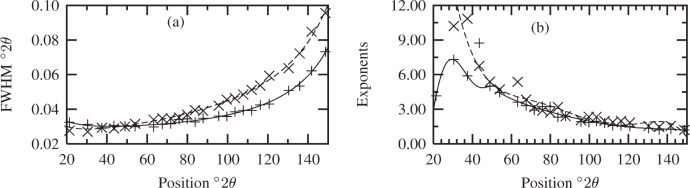
Calibration plots for the split-Pearson VII line profiles, generated from the SRM 660a (LaB_6_) diffraction pattern and used to model the instrument function, *K*(2*θ*). (a) The FWHM vs 2*θ* for low- (+ and solid line) and high- (× and dashed line) angle sides of the peak. (b) The exponents vs 2*θ* for low- (+ and solid line) and high- (× and dashed line) angle sides of the peak.

Diffraction patterns were collected on a Siemens D500^2^ diffractometer equipped with a focusing Ge incident beam monochromator, sample spinner and a scintillation detector. Copper K*_α_*_1_ radiation with a wavelength *λ* = 0.154 059 45 nm was used. The divergence slit was 0.67°, while the receiving optics included a slit of 0.05° and 2° Soller slits. Data were collected in discrete regions straddling the maxima of each profile, with the step and scan width of each region being varied in correspondence with the FWHM. Count times were varied so as to obtain an approximately constant total number of counts for each scan region. The instrument profile function was determined using a split-Pearson VII profile shape function fitted to 22 reflections collected from SRM 660a (LaB_6_). Figure 7, shows the FWHMs and exponents for the split-Pearson VII profile function. The low- and high-FWHMs were fitted using [34],

$${\text{FWHM}}^{2}=A{\text{tan}}^{2}\theta +D{\text{cot}}^{2}\theta +C\text{tan}\;\theta +D,$$ (35)

while the low- and high-exponents were fitted using a fifth-order polynomial.

**Fig. 8:**
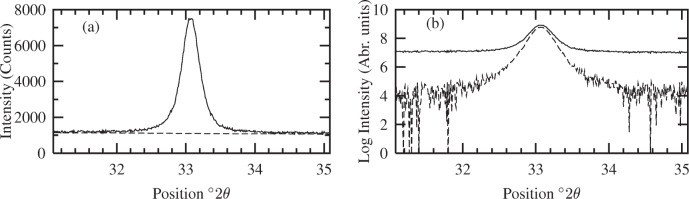
CeO_2_ experimental 200 profile, *g*(2*θ*): (a) The observed 200 profiles (solid line) and estimated background level (dashed line) over (2*θ*_0_ ± 2)°2*θ*, the range over which the analysis was carried out. (b) Logarithm of the 200 measured profile before (solid line) and after the background estimation (dashed line).

The count times for the CeO_2_ data were optimized using Eq. (32) so that the percentage error was kept in the range 1 % to 3 % for all peaks in the CeO_2_ pattern. The scan ranges for the CeO_2_ data were considerably wider, in proportion to the FWHM, than those used for the data collection from SRM 660a. This ensured a reasonable determination of the tails of the profiles and background levels. The CeO_2_ 200 line profile is shown in Fig. 8(a). This illustrates a typical experimental line profile using the above conditions and settings. The estimated (linear) background level is also shown. A log plot of the 200 line before and after the background estimation is shown in Fig. 8(b). The procedure for determining the background level is as described in Sec. 4.1.

### 5.2 TEM Details

Particle agglomerates were gently crushed in ethanol using a mortar and pestle. A portion of the dilute slurry was dispersed on a holey carbon film and left to dry. Once in the TEM, a series of micrographs of particles were taken at a fixed magnification of 200k×. In the *preliminary* examination reported here, these negatives were scanned and analysed by manually approximating the particle size with an oval. The oval’s major and minor axes were adjusted so as to tangentially intersect the particle surface facets.

There are several sources of error in the measurements: TEMs typically have a 5 % error in length scale measurements; also, imaging the particle clusters means that particles are at different heights, which results in Fresnel fringes around the particles making it harder to identify particle edges. Further, larger particles give better contrast and it is easier to detect their edges, so it is possible to inadvertently preferentially choose larger particles over smaller ones.

A frequency histogram for about 850 particles is discussed in Sec. 6.2. It is shown that the Bayesian/MaxEnt size distributions determined from the non-overlapped *hkl* profiles of the CeO_2_ diffraction pattern are in reasonable agreement with TEM data.

## 6. Analysis of CeO_2_ X-Ray Diffraction Data

Two levels of application of the Bayesian and MaxEnt theory has been chosen in our analysis. We refer to these as the *qualitative* and *quantitative* approaches, to reflect their degree of rigor (see Secs. 6.1 and 6.2, respectively).

### 6.1 Qualitative Analysis

The qualitative analysis is used to determine the type and nature of specimen broadening, by first determining the specimen profile, *f*, followed by the application of the Warren-Averbach and Williamson-Hall methods. The integral breadths, from a Williamson-Hall plot, identify the presence of both strain- and size-broadening contributions, while plotting multiple-order Fourier coefficients and all other available Fourier coefficients on the same axes also allows size- and strain-broadening contributions to be identified (see [30]).

We have introduced the fuzzy pixel/MaxEnt method for determining *f* to ensure that no artifacts (such as spurious oscillations in the tails of *f*) are promulgated to the solution, and also to preserve the positivity of *f*.

We stress that unlike traditional methods, the approach in this section makes no assumptions at all about the nature of the specimen profile or broadening (i.e., be it Gaussian, Lorentzian, Voigtian, etc.). Thus, in further distinction from traditional deconvolution approaches, our approach facilitates the subsequent unbiased assessment of anisotropic broadening in the specimen, for example using contrast factors [35].

**Fig. 9:**
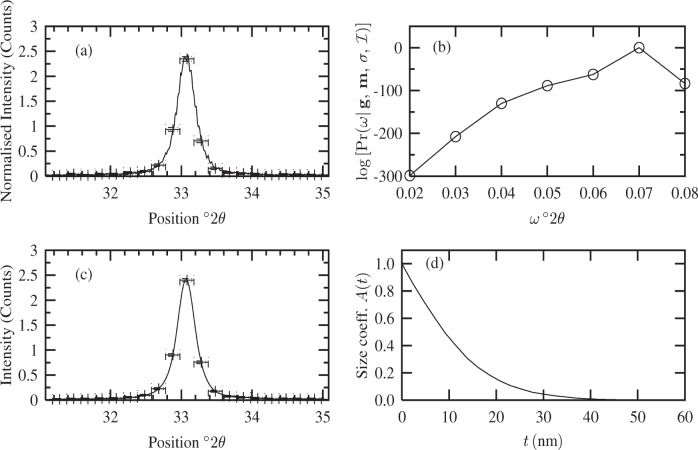
Specimen profiles from the “old” MaxEnt method and fuzzy pixel/MaxEnt method for the measured CeO_2_ 200 line. (a) “Old MaxEnt” specimen profile (solid line + error bars). (b) logPr(ω|g,m,σ,ℐ) distribution to determine the optimum fuzzy pixel width, $\hat{\omega }\approx 0.07{}^{\circ}2\theta$. (c) Fuzzy Pixel/MaxEnt specimen profile (solid line + error bars). (d) Fuzzy Pixel/MaxEnt Fourier coefficients (solid line).

Figure 9 shows an example of the fuzzy pixel/MaxEnt method applied to the CeO_2_ measured 200 line profile given in Fig. 8. Figure 9(a) is an example of the “old” MaxEnt method, showing the effect of noise amplification. On applying the fuzzy pixel/MaxEnt method, the correlation length scale for the profile was determined, as discussed in Sec. 3.7 and is shown in Fig. 9(b); the subsequent *f* and Fourier coefficients for the 200 line profile are given in Figs. 9(c) and (d), respectively. As demonstrated in the analysis of the simulated data, there is noticeable improvement in the quality of the solution line profile using the fuzzy pixel/MaxEnt method. This approach was applied to all the non-overlapped line profiles, including 111, 200, 220, 400, 422, 511, and 531.

**Fig. 10:**
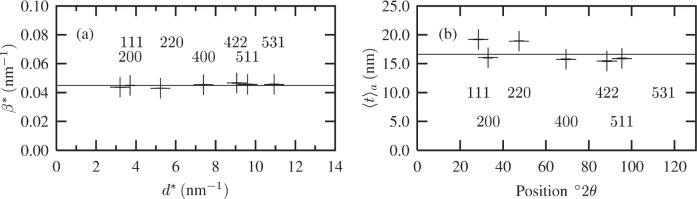
Volume- and area-weighted sizes from the Fuzzy Pixel/MaxEnt line profiles for measured CeO_2_ data. (a) Classical Williamson-Hall plot of the integral breadths, showing no dependency on *hkl*. This suggests that the crystallites are spherical in shape. (b) Area-weighted sizes determined from the Fourier coefficients of the Fuzzy Pixel/MaxEnt line profiles.

The volume- and area-weighted sizes were determined from the Williamson-Hall plot and Fourier coefficients, respectively. These results are shown in Fig. 10 and summarized in Table 3.

Figure 10(a) shows the Williamson-Hall plot for the non-overlapped line profiles. It is evident that size effects are the dominant source of specimen broadening, since there is no detectable slope in the integral breadth data. Moreover, there is no systematic variation of the integral breadths with *hkl*, further suggesting that the crystallite shape is independent of *hkl*. From these results, we can infer that the average shape of the crystallites is spherical. This is further supported by the area-weighted sizes shown in Fig. 10(b). These results were determined by applying Eq. (30) to the Fourier coefficients of the fuzzy pixel/MaxEnt specimen profiles and plotted over the entire 2*θ*-range. Again, the relative uniformity of this plot suggests that size effects are the major source of specimen broadening and that crystallites are near-spherical in shape. Deviations for the 111 and 220 data points in Fig. 10(b) arise from the differentiation of Eq. (30) in the region *t* → 0, where perturbations in the Fourier coefficients cause large changes in the area-weighted size [30]. In addition, the Fourier coefficients for all the non-overlapped *hkl* lines suggest that the maximum crystallite size is ≈50 nm to 60 nm. An example of this can be seen in Fig. 9(d), where *A*(*t*) ≈ 0 for ≈50 nm to 60 nm. This can also be seen from the discussion in Sec. 2.2 and by inspecting Fig. 1, where the boundary conditions for *A*(*t*) [or *V*(*t*)] are defined in terms of the maximum size in the direction of the scattering vector.

Referring to Table 3, a spherical crystallite shape model was used to determine the area- and volume-weighted diameters, together with Eqs. (29a) and (29b), respectively. The log-normal distribution parameters, *D*_0_, *σ*_0_, 〈*D*〉, and $\sigma_{\langle D\rangle }^{2}$ were determined using the equations developed by Krill and Birringer [1] and Eq. (28), which relate the log-normal parameters to the area- and volume-weighted sizes and the average diameter, 〈*D*〉, and variance *σ*_〈_*_D_*_〉_.

It can be seen from Fig. 9 and Table 3, that the area and volume-weighted sizes are relatively uniform for the 2*θ* (or *hkl*) range. The quoted uncertainty for the averages was determined from a sum of least squares analysis of the uncertainties in the tabulated results.

The average results for *D*_0_ and *σ*_0_, were used to define a log-normal *a priori* model in the Bayesian/MaxEnt method (see Sec. 6.2). By defining the *a priori* model as a log-normal distribution, we are essentially testing the assumption that the size distribution is log-normal.

If the underlying size distribution is indeed log-normal, with parameters close to those in Table 3, then we would expect the Bayesian/MaxEnt solution to lie “close” to the *a priori* model. However, if the Bayesian/MaxEnt solution were “some distance” from the *a priori* model, this would imply that either the underlying parameters or the model were inappropriately defined. The former case was demonstrated in analysis of the simulated data (see Sec. 4.3), where uncertainties in the log-normal model were passed onto the Bayesian/MaxEnt solution; the latter case requires additional Bayesian analysis to test possible models [36,37].

In summary, the qualitative analysis has applied the fuzzy pixel/MaxEnt method to determine the specimen profile *f* for all non-overlapped line profiles from the CeO_2_ measured data (see Fig. 9). This enabled subsequent analyses to determine the Fourier coefficients, integral breadths, and the area- and volume-weighted sizes. Fig. 10 and Table 3 clearly indicate that the CeO_2_ specimen on average consists of spherical crystallites. While a log-normal distribution can be fitted to these results, a quantitative method such as the Bayesian/MaxEnt technique is needed to determine the CeO_2_ size distribution *directly* from the experimental data and to verify the assumption of a log-normal model.

### 6.2 Quantitative Analysis

The quantitative analysis method uses the *a priori* information determined from the qualitative analysis and the available experimental data (such as the instrument and profile kernels, statistical uncertainties and experimental line profiles) to directly determine the crystallite size distribution.

The MaxEnt method also enables an *a priori* model to be included, while quantifying the uncertainty in the solution size distribution.

In this section, we apply the Bayesian/MaxEnt method to the CeO_2_ data. The analysis presented here follows the steps discussed in Sec. 4.3. Two *a priori* models are used: (i) a uniform model, and (ii) the log-normal distribution determined in Sec. 6.1. The Bayesian/MaxEnt size distributions for each case are fitted with a log-normal distribution, while the size distributions from (ii) are compared with the TEM size distribution, with very good agreement.

#### 6.2.1 Uniform Model

**Fig. 11:**
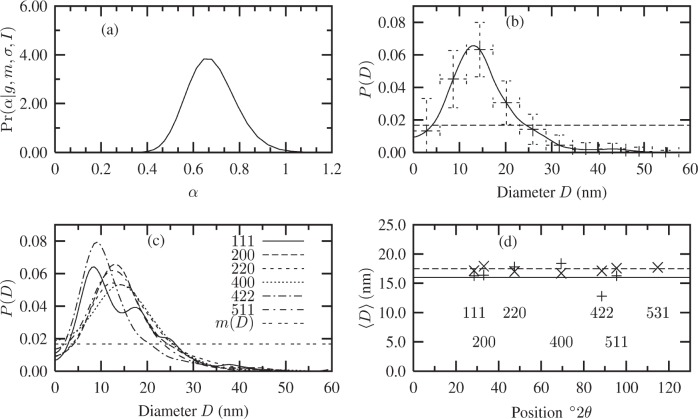
CeO_2_ Bayesian/MaxEnt crystallite-size distribution using a uniform *a priori* model over *D* ∈ [0,60] nm: (a) The Pr(α|g,m,σ,ℐ) distribution, Eq. (17), used to average over the set of solutions, {***P***(*α*)} for the 200 line profile; (b) Bayesian/MaxEnt crys-tallite-size distribution (solid line + error bars) and the *a priori* model over D ∈ [0,60] nm; (c) CeO_2_ Bayesian/MaxEnt crystallite-size distributions for the various CeO_2_ *hkl* profiles; (d) Average diameters from the uniform model (+) and log-normal models (×). The horizontal lines represent the average for each model.

A uniform model was defined over the region *D* ∈ [0,60] nm determined by the Fourier coefficients of the specimen profile, where *A*(*t*) ∼ 0. This is illustrated by the Fourier coefficients for the 200 line profile, given in Fig. 9(d). The Bayesian/MaxEnt size distributions using this model are shown in Fig. 11 for the 200 line profile [see Figs. 11(a and b)]. The size distributions for the non-overlapped line profiles are given in Figs. 11(c and d).

The uncertainties in the Bayesian/MaxEnt size distribution for the 200 line profile indicate how little useful *a priori* information has been transferred from the uniform model to the final distribution. We also notice that the final distribution is some distance from the model, illustrating that the underlying CeO_2_ crystallite size distribution consists of a non-uniform structure. As can be seen in Fig. 11(c), the size distributions are poorly defined in the range of *D* ∈ [0,5] nm, while for *D* ≳ 5 nm the non-uniform structure is evident. Since the size distribution is the only invariant quantity, we also expect the solution for each *hkl* to be the same. From the size distributions given in Fig. 11(c), there is a broad agreement between the distributions, with the exception of the 111 and 422 cases. Both of these distributions are more likely to be susceptible to large experimental uncertainties.

The Bayesian/MaxEnt size distributions were fitted with a log-normal model and the *D*_0_, *σ*_0_, 〈*D*〉, and $\sigma_{\langle D\rangle }^{2}$ parameters determined. These results are given in Table 4. The uncertainties in the solution distributions for the uniform model are also reflected in the uncertainties in the fitted quantities. This is especially the case for the variance of the size distributions, $\sigma_{\langle D\rangle }^{2}$, with an error of ≈80 %. This large uncertainty is a consequence of the scatter of size distributions shown in Fig. 11(c). Such scatter is also noticeable when the average diameters, 〈*D*〉, (Table 4) are plotted, as shown in Fig. 11(d). The average values for *D*_0_, *σ*_0_, 〈*D*〉, and $\sigma_{\langle D\rangle }^{2}$ are again in broad agreement with results determined in Sec. 6.1, once the uncertainties are taken into account.

In summary, the use of the uniform model in the Bayesian/MaxEnt method has shown that there is a non-uniform structure to the CeO_2_ size distributions. However, the lack of information in this model results in large uncertainties and considerable scatter of the distributions when plotted on the same axes [see Fig. 11(c)].

#### 6.2.2 Log-Normal Model

The parameters for the log-normal distribution determined in Sec. 6.1 were used as the non-uniform *a priori* model in the Bayesian/MaxEnt method. The model was defined over the range of *D* ∈ [0,60] nm.

**Fig. 12:**
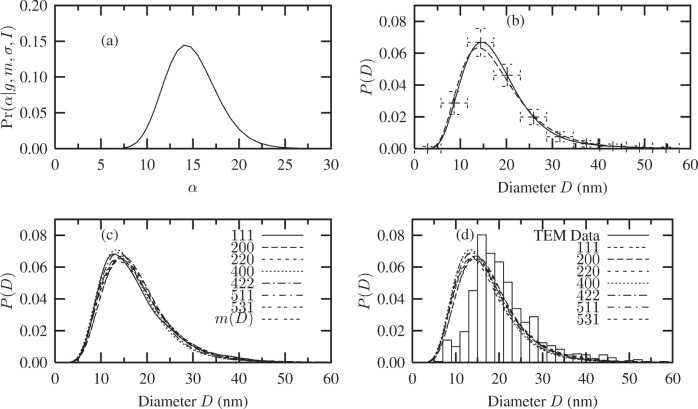
CeO_2_ Bayesian/MaxEnt crystallite-size distribution using a log-normal *a priori* model over *D* ∈ [0,60] nm. (a) Pr(α|g,m,σ,ℐ) distribution, Eq. (17), used to average over the set of solutions {***P***(*α*)}, for the 200 line. (b) Bayesian/MaxEnt crystallite-size distribution (solid line + error bars) and the *a priori* model (dashed line) over D ∈ [0,60] nm. (c) CeO_2_ Bayesian/MaxEnt crys-tallite-size distributions for the non-overlapped line profiles. (d) Comparison of TEM and Bayesian/MaxEnt distributions. Due to difficulties in de-aggregating the CeO_2_ particles and identifying smaller crystallites, the TEM size distribution is poorly defined for *D* ≤ 14 nm. However, for *D* ≥ 14 nm, the comparison is excellent.

The Bayesian/MaxEnt size distributions for this model are shown in Fig. 12. The results are listed in Table 5. Figures 12(a) and (b) show the results for the 200 size distribution using this model. The Bayesian/MaxEnt solution lies close to the log-normal model, while the uncertainties have decreased considerably compared with the size distribution (using a uniform model) in Fig. 11(b); however, although the vertical error bars have decreased, they are still considerable. This can be explained in terms of the influence of the peak-to-background ratio. As discussed in Sec 4.2, the variance of the experimental data is determined by two terms, the statistical noise and the variance in the estimated background level. If the peak-to-background ratio is large (≳10), then the statistical noise dominates and the corresponding error bars in the Bayesian/MaxEnt distribution become small when the solution is close to the underlying size distribution. This has been demonstrated using computer simulations. However, if the peak-to-background ratio is finite (<10), the corresponding error bars in the MaxEnt/Bayesian solution remain finite regardless of how close the solution is to the underlying distribution. This is a consequence of determining the size distribution directly from the experimental data.

The Bayesian/MaxEnt size distributions for all the non-overlapped *hkl* line profiles are shown in Fig. 12(c). They lie very close to each other, reflecting the invariance of the size distribution and remaining close to the log-normal model. The scatter in the size distributions that was noticeable in Fig. 11(c) for the uniform model has disappeared. Further, these results imply that the underlying size distribution from the CeO_2_ crystallites can be described by a log-normal distribution. Comparing these results with the TEM size distribution, very good agreement is obtained for 14 ≲ *D* ≲ 60 nm. Due to its poor statistics, the TEM size distribution is ill-defined for *D* ≲ 14 nm. As mentioned above, the CeO_2_ agglomerates were not separated, making it difficult to identify the smaller crystallites and contributing to the poorly defined region for *D* ≲ 14 nm. The TEM size distribution, given in Fig. 12(d), represents a preliminary set of data.

The correspondence between the Bayesian/MaxEnt size distributions and the TEM distribution is very good for *D* ≥ 14 nm. The size distributions shown in Fig. 12(c) were fitted with a log-normal distribution and the *D*_0_, *σ*_0_, 〈*D*〉, and $\sigma_{\langle D\rangle }^{2}$ parameters were determined. These results are shown in Table 5. The fitted distribution compared very closely with the solution distribution. The small uncertainties in the fitted quantities of Table 5 reflect the quality of the Bayesian/MaxEnt distributions. This can also be seen in the low uncertainty in the variance, $\sigma_{\langle D\rangle }^{2}$, which is ≈8 %.

The average quantities given in Table 5 can be considered to represent the size distribution for the CeO_2_ specimen. Hence, the use of the fuzzy pixel/Bayesian/MaxEnt methods has determined the specimen profile, *f*, and enabled size effects to be identified as the major source of specimen broadening. The analysis of the line profiles has shown that the crystallite shape is spherical, on average. The Fourier coefficients of the specimen profiles also show that the crystallites have a maximum size of ≈60 nm. This was subsequently shown from the Bayesian/MaxEnt size distributions. Using this information, the Bayesian/MaxEnt method successfully determined the CeO_2_ size distribution. While the size distributions using a uniform *a priori* model broadly agree with the results from the fuzzy pixel analysis, the uncertainty in the results is large; on using a log-normal *a priori* model, considerable improvements in the size distribution were obtained. The non-uniform structure in the model has been transferred to the Bayesian/MaxEnt solution.

**Fig. 13:**
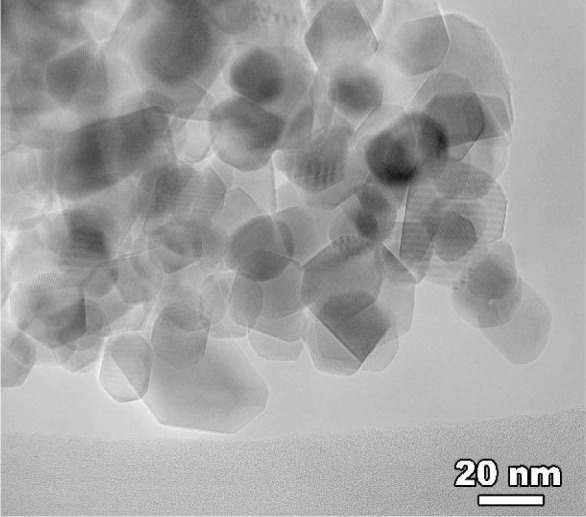
A TEM micrograph of the CeO_2_ specimen taken at a magnification of 200k×. The crystallites appear to have a spherical-like shape and size that are in the range predicted by the crystallite-size analysis presented here.

The TEM micrograph of the CeO_2_ specimen, shown in Fig. 13, confirms the results that have been determined from the x-ray diffraction data. From the micrograph, it can be seen that the crystallites are near-spherical in shape. It can also be seen that the crystallites are in the range of size predicted by crystallite-size analysis. Considerable overlapping of the crystallites, which complicates the task of gathering sufficiently reliable data for the TEM size distribution is evident.

## 7. Conclusion

The central aim of this study was to develop a single-step, self-contained method for determining the crystal-lite-size distribution and shape from experimental line profile data. We have shown that the crystallite-size distribution can be determined without assuming a functional form for the size distribution, determining instead the size distribution with the least assumptions.

This was achieved by reviewing size broadening theory showing how the observed line profile can be expressed in terms of the instrument kernel, line profile kernel and size distribution. It was also shown that the instrument and line profile kernels could be combined into a single kernel, hence enabling the simultaneous removal of instrumental broadening while determining the size distribution (see Sec. 2).

The development of this method made use of two fundamental observations—that distributions such as the specimen profile and size distribution must be *both* positive and additive. Drawing on extensive theoretical developments, the entropy function was selected as the function that can attribute values to the specimen line profile and size distribution, while preserving the positivity and additivity of the profile and distribution. It can be also argued that the entropy function is the only function that produces consistent results in the light of experimental data (see Sec. 3.2).

Using the mathematical and statistical foundations of Bayesian theory, the *a posteriori* distributions of *P*(***D***) in terms of the experimental data, statistical noise and scattering kernel can be determined. By maximizing this distribution, the most probable size distribution can be calculated from the experimental line profile, without making any assumptions concerning the functional form of the size distribution. Determining the most probable size distribution addresses the inherent non- uniqueness and ill-conditioning in the integral equations arising from scattering and instrumental broadening. The generality of this formalism enables any crys-tallite shape to be used and any number of principal axes, ***D*** = {*D*_1_, *D*_2_, *D*_3_}, of the crystallite shape can be included in determining the corresponding size distributions.

Simulated data were used to test the fuzzy pixel and Bayesian/MaxEnt methods on size-broadened line profiles. The reliability of these methods was established by showing that they can reproduce the underlying parameters of the area- and volume-weighted sizes, and the parameters of the size distributions.

The application of these methods to CeO_2_ experimental data generally produced very good results. The line profile analysis applying fuzzy pixel/MaxEnt methods produced reliable and consistent results over a wide range of low-, mid- and high-angle profiles.

The application of the Bayesian/MaxEnt method to the CeO_2_ data demonstrated that this method can determine size distributions, while making the minimum number of assumptions. The use of a uniform *a priori* model produced broadly consistent results with the fuzzy pixel/MaxEnt method; however, the lack of information defined in this model was evident in the large uncertainties of the estimated quantities.

Using the fuzzy pixel/MaxEnt results as the log-normal *a priori* model demonstrated that once “useful” information is encoded in the model, improvements in the size distributions and considerable reduction in the uncertainties can be achieved. Analysis of the x-ray diffraction profiles using the log-normal model in the Bayesian/MaxEnt method revealed that the crystallites are spherical in shape, with a size distribution corresponding to the distribution in Fig. 12 and average quantities in Table 5. The comparison of these Bayesian/MaxEnt results with TEM results is favorable, but it does reveal shortcomings in the collected TEM data arising from particle aggregation. The TEM distribution micrographs support the results from the line profile analysis.

The use of simulated and experimental data demonstrates that the fuzzy pixel/Bayesian/MaxEnt methods are fully quantitative in their ability to determine and attribute errors to the solution line profiles and size distributions.

Although the results from the Bayesian/MaxEnt method are in good agreement and address the limitations of the earlier work (see [30,10]), several important issues have been raised and are the subject of further investigation. These concern the accurate background estimation of the observed line profile and are very important; for example, the analysis of simulated data demonstrated how systematic errors affect the Fourier coefficients. Recently, David and Sivia [38] have developed a Bayesian technique for estimating the background, which can be adopted in this method. Another problem encountered was in the estimation and quantifying of a non-uniform *a priori* model. In this analysis we have used the information determined from the fuzzy pixel/MaxEnt method; however, the issue of determining the *a priori* model can also be addressed in a Bayesian context, by using a process of model selection [36,37] and defining an *a posteriori* distribution of parameters in the model [20]. Further, only single line profiles were analyzed here; while the formalism has been expressed for overlapped line profiles, demonstrating that the Bayesian/MaxEnt method is flexible in its application, additional analysis of overlapped line profiles is needed.

The literature has seen considerable debate over the type of distribution that best describes the distribution of sizes (see [1, 2, 39, 40]). In the analysis presented here we have simply used a log-normal distribution to demonstrate that the Bayesian/MaxEnt method can reproduce the parameters. Moreover, the position we have taken in developing the Bayesian/MaxEnt method is that we are not concerned with the type of distribution; rather, we have produced a reliable and consistent method that can determine the specimen profile and/or the size distribution, given our understanding of the experimental data, statistical noise and instrumental effects.

## Figures and Tables

**Table 1:** Area- and volume-weighted sizes for the 200 and 400 specimen line profiles (*f*) from the “old” MaxEnt and fuzzy pixel/MaxEnt methods. 〈*D*〉_a_ and 〈*D*〉_v_ values were determined using Eq. (29). The *D*_0_, *σ*_0_, 〈*D*〉, and $\sigma_{\langle D\rangle }^{2}$ values were determined from Eq. (28). The percentage differences between the calculated and theoretical values are given in parentheses

Results	“old” MaxEnt		Fuzzy Pixel/MaxEnt	
	200	400	200	400
*R_f_*(%)	4.2	10.9	2.7	3.1
*R*_w_(%)	2.9	3.7	3.0	3.7
〈*t*〉_a_(nm)	19.9 ± 0.1(13.3 %)	20.4 ± 0.1(16.0 %)	17.89 ± 0.07(1.9 %)	20.5 ± 0.1(16.5 %)
〈*D*〉_a_(nm)	29.8 ± 0.2	30.5 ± 0.2	26.8 ± 0.1	307 ± 0.2
〈*t*〉_v_(nm)	26.63 ± 0.07(1.7 %)	28.0 ± 0.2(7.1 %)	25.86 ± 0.04(1.2 %)	27.4 ± 0.2(4.8 %)
〈*D*〉_v_(nm)	35.51 ± 0.09	37.4 ± 0.2	34.48 ± 0.05	36.6 ± 0.3
*D*_0_(nm)	19.3 ± 0.4(48.1 %)	18.4 ± 0.5(41.5 %)	14.3 ± 0.2(10.1 %)	19.8 ± 0.6(52.0 %)
*σ*_0_	1.52 ± 0.01(10.7 %)	1.57 ± 0.02(7.8 %)	1.650 ± 0.007(3.0 %)	1.52 ± 0.02(10.6 %)
〈*D*〉(nm)	21.06 ± 0.43(40.4 %)	20.4 ± 0.5(36.0 %)	16.3 ± 0.2(8.4 %)	2.6 ± 0.6(44.1 %)
$\sigma_{\langle D\rangle }^{2}\left({\text{nm}}^{2}\right)$	84 ± 5(15.3 %)	93 ± 7(27.2 %)	75 ± 3(2.9 %)	89 ± 8(22.4 %)

**Table 2:** *P*(*D*) results from the Bayesian/MaxEnt method for the 200 and 400 line profiles using different *a priori* models. The values for *D*_0_, *σ*_0_, 〈*D*〉, and $\sigma_{\langle D\rangle }^{2}$ were determined by fitting the Bayesian/MaxEnt solutions with a log-normal distribution. The percentage difference between calculated and theoretical values are given in parentheses

Results	Uniform model		“Low” res. model	
	200	400	200	400
*R_f_*(%)	23.0	40.0	22.2	19.1
*D*_0_(nm)	13.9 ± 0.3(6.5 %)	11.9 ± 0.9(8.8 %)	14.8 ± 0.2(13.4 %)	12.5 ± 0.2(4.4 %)
*σ*_0_	1.589 ± 0.003(6.5 %)	2.14 ± 0.03(25.8 %)	1.612 ± 0.002(5.1 %)	1.544 ± 0.002(9.2 %)
〈*D*〉(nm)	15.5 ± 0.3(3.0 %)	15 ± 2(5.8 %)	16.6 ± 0.2(10.4 %)	13.7 ± 0.2(8.7 %)
$\sigma_{\langle D\rangle }^{2}$ (nm^2^)	57 ± 3(22.0 %)	197 ± 145(>100 %)	70 ± 2(3.9 %)	39 ± 1(46.7 %)

**Table 3:** Summary of CeO_2_ data analysis. The area- and volume-weighted sizes were determined from the specimen profile of the fuzzy pixel/MaxEnt method. The 〈*t*〉_a_ and 〈*t*〉_v_ results were determined directly from *f* using Eqs. (30) and (31), respectively. The area- and volume-weighted diameters were determined using Eq. (29), while the log-normal parameters were determined from Krill and Birringer [19] and using Eq. (28)

*hkl*	〈*t*〉_a_ (nm)	〈*D*〉_a_ (nm)	〈*t*〉_v_ (nm)	〈*D*〉_v_ (nm)	*D*_0_ (nm)	*σ*_0_	〈*D*〉 (nm)	$\sigma_{\langle D\rangle }^{2}$ (nm^2^)
111	19.21 ± 0.05	28.81 ± 0.07	22.88 ± 0.03	30.50 ± 0.04	25.0 ± 0.2	1270 ± 0.007	25.7 ± 0.2	39 ± 2
200	16.04 ± 0.06	24.06 ± 0.08	22.22 ± 0.05	29.63 ± 0.06	14.3 ± 0.2	1.578 ± 0.007	15.9 ± 0.2	58 ± 2
220	18.92 ± 0.04	28.38 ± 0.06	23.24 ± 0.04	31.00 ± 0.05	22.8 ± 0.2	1.345 ± 0.006	23.8 ± 0.2	52 ± 2
400	15.76 ± 0.06	23.64 ± 0.09	22.03 ± 0.11	29.4 ± 0.2	13.7 ± 0.3	1.59 ± 0.01	15.3 ± 0.3	56 ± 3
422	15.45 ± 0.08	23.2 ± 0.1	21.5 ± 0.1	28.6 ± 0.1	13.7 ± 0.3	1.58 ± 0.01	15.2 ± 0.3	54 ± 3
511	15.91 ± 0.07	23.9 ± 0.1	21.9 ± 0.1	29.2 ± 0.2	14.4 ± 0.3	1.57 ± 0.01	16.0 ± 0.3	57 ± 3
531	15.04 ± 0.04	22.55 ± 0.07	21.9 ± 0.1	29.20 ± 0.16	11.8 ± 0.2	1.66 ± 0.01	13.5 ± 0.2	53 ± 3
Average	16.6 ± 0.2	24.9 ± 0.2	22.2 ± 0.2	29.6 ± 0.3	16.5 ± 0.6	1.51 ± 0.03	17.9 ± 0.7	53 ± 7

**Table 4:** Size distribution results using a uniform *a priori* model in the Bayesian/MaxEnt method. The Bayesian/MaxEnt size distributions given in Fig. 11(c) were fitted with a log-normal size distribution and the above parameters determined

*hkl*	*D*_0_ (nm)	*σ*_0_	〈*D*〉 (nm)	$\sigma_{\langle D\rangle }^{2}$ (nm^2^)
111	13.1 ± 0.4	1.9 ± 0.1	16.3 ± 0.8	140 ± 43
200	14.7 ± 0.3	1.61 ± 0.03	16.4 ± 0.4	69 ± 7
220	15.5 ± 0.4	1.70 ± 0.08	17.8 ± 0.6	104 ± 25
400	15.8 ± 0.8	1.7 ± 0.1	18 ± 1	120 ± 48
422	11.1 ± 0.2	1.728 ± 0.007	12.8 ± 0.2	58 ± 2
511	14.3 ± 0.3	166 ± 0.05	16.3 ± 0.4	78 ± 15
Average	14 ± 1	1.7 ± 0.2	16 ± 2	95 ± 71

**Table 5:** Size distribution results using a log-normal *a priori* model in the Bayesian/MaxEnt method. The Bayesian/MaxEnt size distributions given in Fig. 11(c) were fitted with a log-normal size distribution and the above parameters determined

*hkl*	*D*_0_ (nm)	*σ*_0_	〈*D*〉 (nm)	$\sigma_{\langle D\rangle }^{2}$ (nm^2^)
111	15.9 ± 0.2	1.50 ± 0.008	17.2 ± 0.2	54 ± 2
200	16.64 ± 0.04	1.469 ± 0.005	17.91 ± 0.04	51±1
220	15.68 ± 0.06	1.502 ± 0.008	17.04 ± 0.08	52 ± 2
400	15.48 ± 0.01	1.480 ± 0.005	16.72 ± 0.03	46 ± 1
422	15.86 ± 0.07	1.4799 ± 0.0002	17.12 ± 0.08	48.7 ± 0.5
511	16.21 ± 0.07	1.497 ± 0.002	17.58 ± 0.08	54.7 ± 0.6
531	16.32 ± 0.07	1.500 ± 0.005	17.72 ± 0.09	56 ± 1
Average	16.0 ± 0.2	1.49 ± 0.01	17.3 ± 0.3	52 ± 3

## Footnotes

1 Taking Eq. (32), we see that in the limit of *R*_pb_ → 1, *σ*_p_ → ∞. On the other hand, in the limit of *R*_pb_ → ∞, $\sigma_{p}\to 1/\sqrt{I_{\max,\text{bg}}}$. For example, with *R*_pb_ ≈ 15, $\sigma_{p}\approx 1.2/\sqrt{I_{\max,\text{bg}}}$.

2 Certain commercial equipment, instruments, or materials are identified in this paper to foster understanding. Such identification does not imply recommendation or endorsement by the National Institute of Standards and Technology, nor does it imply that the materials or equipment identified are necessarily the best available for the purpose.
